# Supplementary figures and images for: Guanidine production by plant homoarginine-6-hydroxylases
Source: eLife. 2024 Apr 15;12:RP91458. doi: 10.7554/eLife.91458 (PMC11018352; doi:10.7554/eLife.91458)

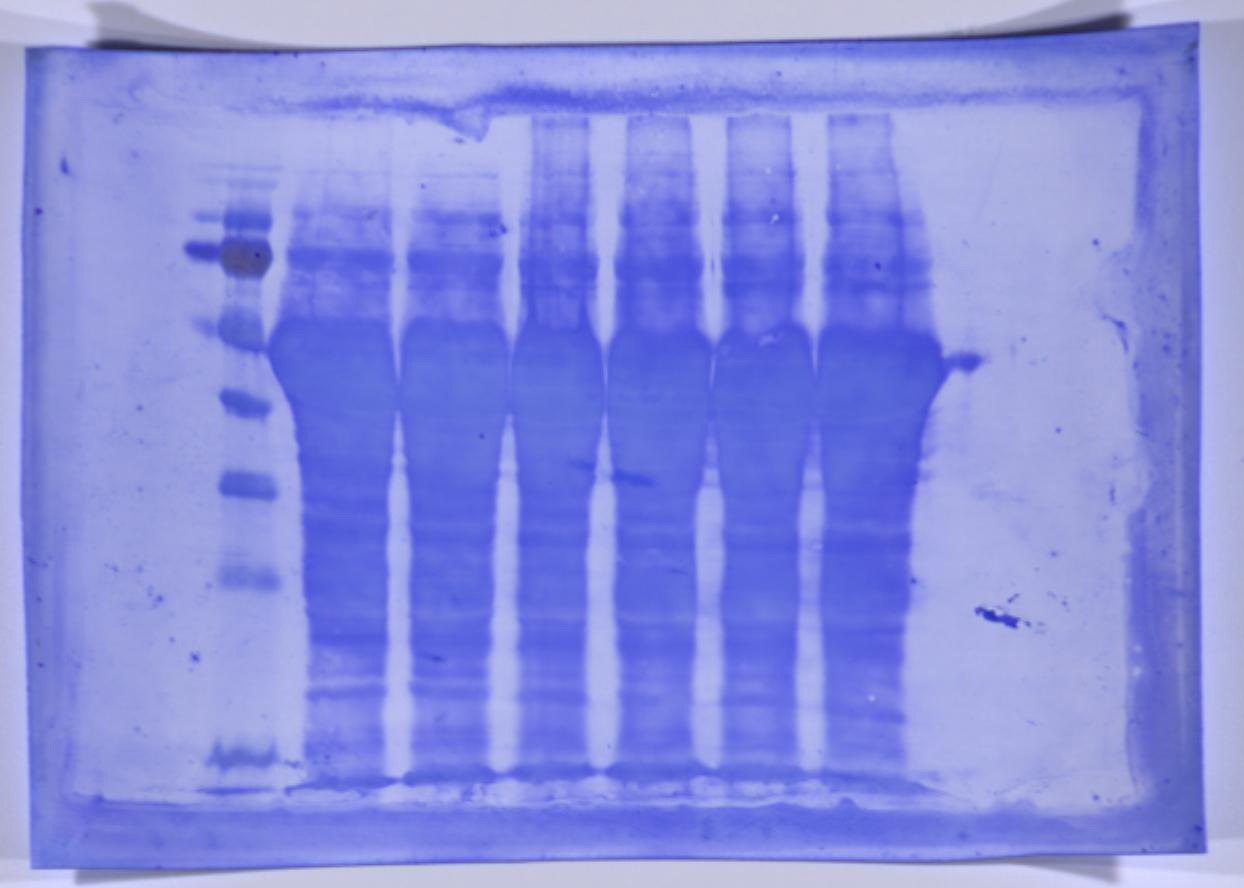

Supplement: Figure 4—figure supplement 1—source data 1. [file elife-91458-fig4-figsupp1-data1.zip › 91458R1 Figure 4ΓÇöfigure supplement 1ΓÇösource data 1/231018 EFE-GFP 2023.10.18_16.22.10_Co.jpg]

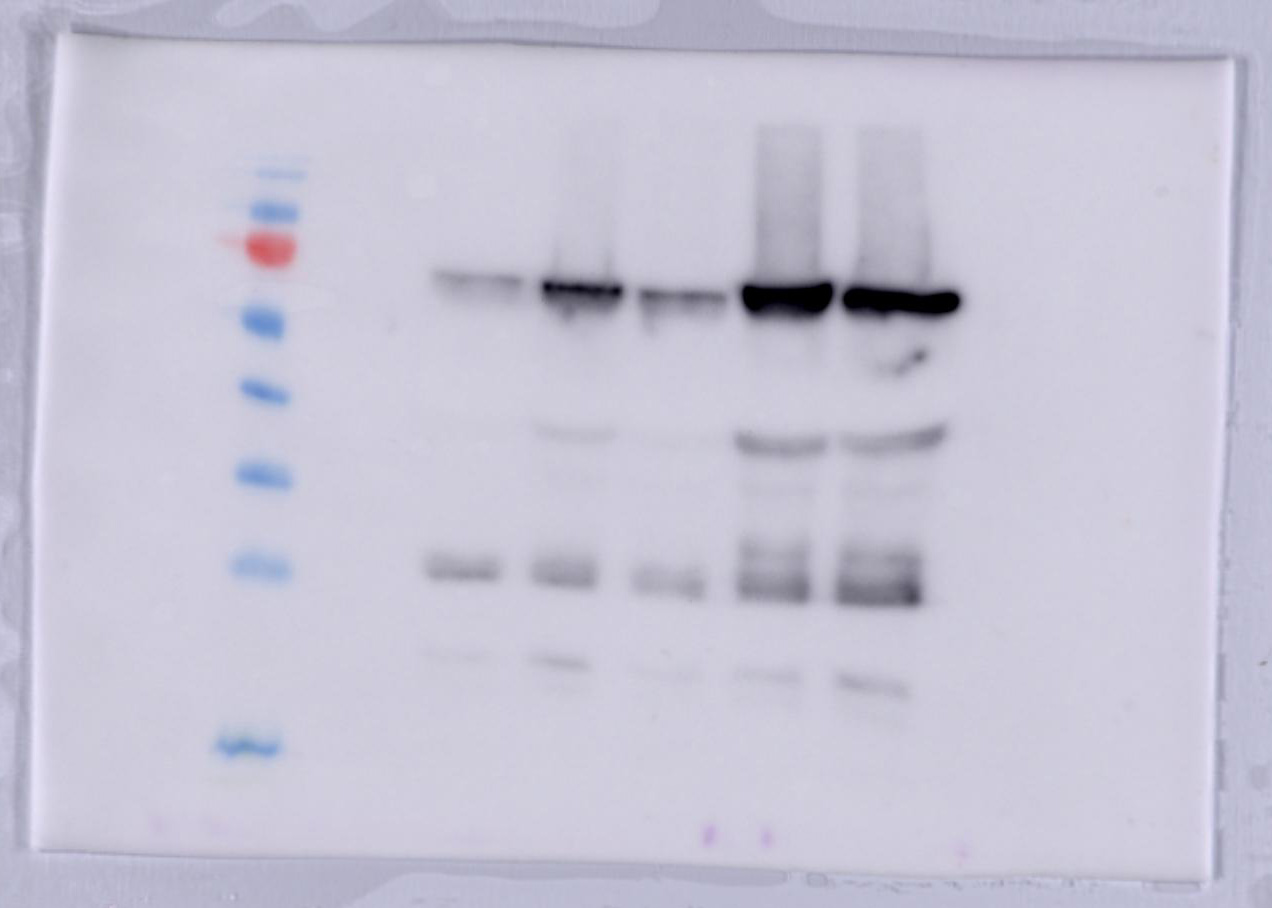

Supplement: Figure 4—figure supplement 1—source data 1. [file elife-91458-fig4-figsupp1-data1.zip › 91458R1 Figure 4ΓÇöfigure supplement 1ΓÇösource data 1/231017EFE-GFP 15min 2023.10.17_14.43.57_Ch+Marker.jpg]

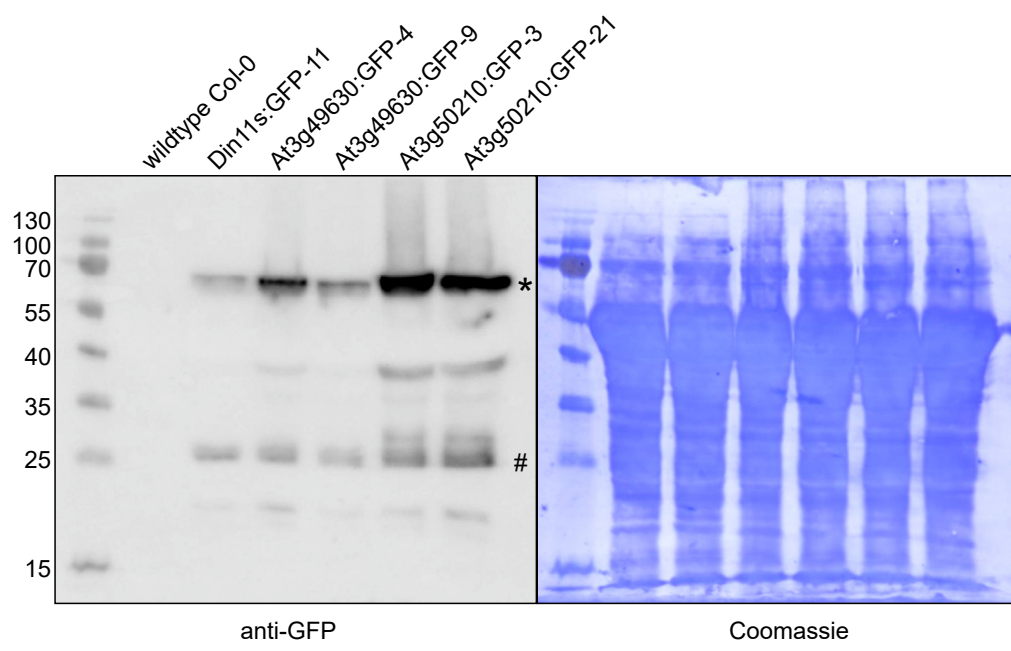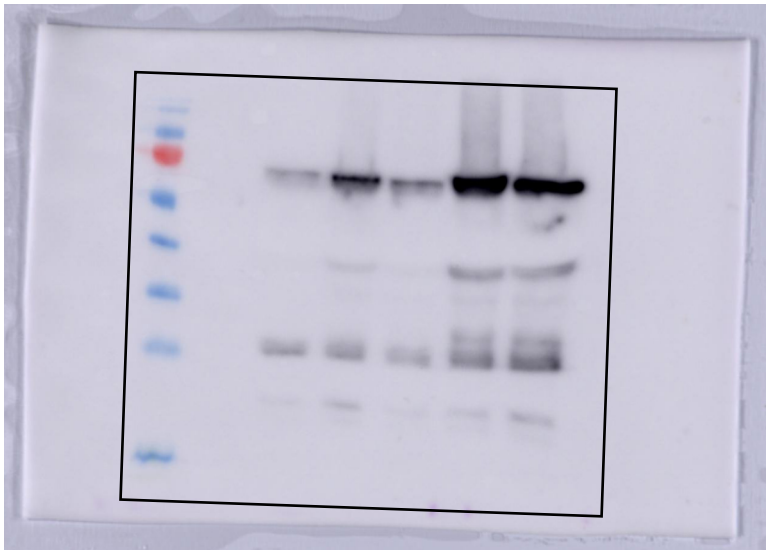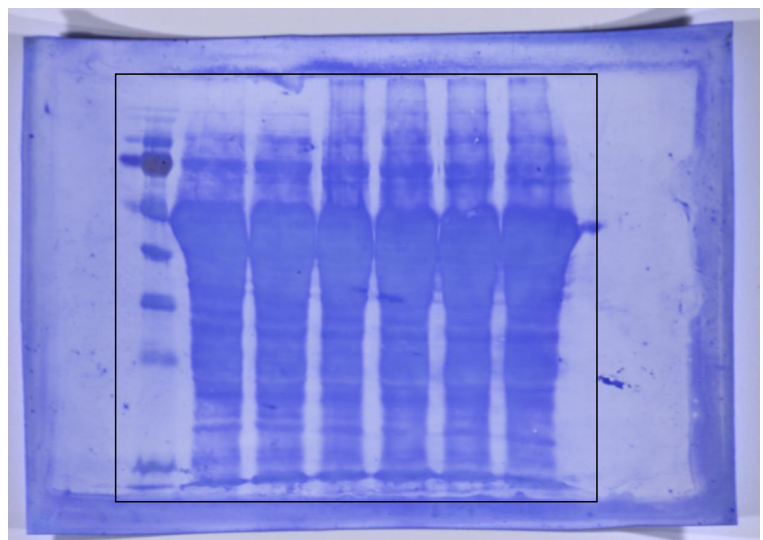

Supplement: Figure 4—figure supplement 1—source data 2. [file elife-91458-fig4-figsupp1-data2.pdf]
