# Supplementary material for: Guanidine production by plant homoarginine-6-hydroxylases
Source: eLife. 2024 Apr 15;12:RP91458. doi: 10.7554/eLife.91458 (PMC11018352; doi:10.7554/eLife.91458)
Supplement: Supplementary file 1. [file elife-91458-supp1.docx]

**Supplementary file 1: Kinetic constants of Arabidopsis 2-ODD-C23 enzymes**

|  | Homoarginine variable, 0.5 mM 2-oxoglutarate | | |
| --- | --- | --- | --- |
|  | Inhibitor  (1 mM) | A_max_  (nmol s^-1^ mg^-1^) | *K*_M_  (mM) |
| Din11s | none | 13.4 ±3.2 | 1.9 ±0.3 |
|  | Arginine | 12.9 ±3.5 | 3.6 ±0.5 |
|  | Canavanine | 11.2 ±2.7 | 5.5 ±1.0 |
| At3g49630 | none | 5.6 ±1.2 | 4.6 ±0.2 |
|  | Arginine | 5.4 ±1.3 | 4.1 ±0.8 |
|  | Canavanine | 5.4 ±1.4 | 4.1 ±0.7 |
| At3g50210 | none | 20.9 ±1.7 | 0.78 ±0.15 |
|  | Arginine | 21.3 ±3.7 | 1.75 ±0.43 |
|  | Canavanine | 16.2 ±2.3 | 0.74 ±0.09 |

|  | Arginine variable, 0.5 mM 2-oxoglutarate | | 2-Oxoglutarate variable, 10 mM homoarginine | | |
| --- | --- | --- | --- | --- | --- |
|  | A_max_  (nmol s^-1^ mg^-1^) | *K*_M_  (mM) | A_max_  (nmol s^-1^ mg^-1^) | *K*_M_  (µM) | *K*_I_  (mM) |
| Din11s | 3.1 ±1.4 | 6.0 ±5.1 | 5.2 ±0.9 | 30.1 ±9.4 | 17.6 ±9.6 |
| At3g49630 | n.d. |  | 3.1 ±0.4 | 53.8 ±37.1 | 36.8 ±8.8 |
| At3g50210 | n.d. |  | 19.0 ±7.2 | 69.7 ±23.2 | 35.3 ±20.1 |

All data are the average ±SD from 3 to 5 independent enzyme preparations
