# Supplementary material for: Guanidine production by plant homoarginine-6-hydroxylases
Source: eLife. 2024 Apr 15;12:RP91458. doi: 10.7554/eLife.91458 (PMC11018352; doi:10.7554/eLife.91458)
Supplement: Supplementary file 2. [file elife-91458-supp2.docx]

**Supplementary file 2: Arabidopsis T-DNA insertion lines used in this study**

| Line | NASC stock Nr. | Position of T-DNA | Reference |
| --- | --- | --- | --- |
| Col-0 | N60000 | wildtype | (Alonso et al., 2003) |
| SALK_071632 | N571632 | 3^rd^ intron of *Din11* | (Alonso et al., 2003) |
| GK-363E06 | N434806 | 1^st^ exon of *Din11L* 5′-UTR of *Din11s* | (Kleinboelting et al., 2012) |
| SALK_118391 | N618391 | 4^th^ intron of *At3g49630* | (Alonso et al., 2003) |
| SAIL_512_A07 | N821581 | 6^th^ intron of *At3g49630* | (Sessions et al., 2002) |
| SALK_095112 | N595112 | 7^th^ exon of *At3g49630* | (Alonso et al., 2003) |
| SALK_133126 | N633126 | 3^rd^ exon of *At3g50210* | (Alonso et al., 2003) |
| SALK_098742 | N598742 | 1^st^ intron of *At3g50210* | (Alonso et al., 2003) |

**References:**

Alonso, J. M., Stepanova, A. N., Leisse, T. J., Kim, C. J., Chen, H., Shinn, P., Stevenson, D. K., Zimmerman, J., Barajas, P., Cheuk, R., Gadrinab, C., Heller, C., Jeske, A., Koesema, E., Meyers, C. C., Parker, H., Prednis, L., Ansari, Y., Choy, N., . . . Ecker, J. R. (2003). Genome-wide insertional mutagenesis of *Arabidopsis thaliana*. *Science*, *301*(5633), 653-657. <https://doi.org/10.1126/science.1086391>

Kleinboelting, N., Huep, G., Kloetgen, A., Viehoever, P., & Weisshaar, B. (2012). GABI-Kat SimpleSearch: new features of the *Arabidopsis thaliana* T-DNA mutant database. *Nucleic Acids Research*, *40*(Database issue), D1211-1215. <https://doi.org/10.1093/nar/gkr1047>

Sessions, A., Burke, E., Presting, G., Aux, G., McElver, J., Patton, D., Dietrich, B., Ho, P., Bacwaden, J., Ko, C., Clarke, J. D., Cotton, D., Bullis, D., Snell, J., Miguel, T., Hutchison, D., Kimmerly, B., Mitzel, T., Katagiri, F., . . . Goff, S. A. (2002). A high-throughput Arabidopsis reverse genetics system. *Plant Cell*, *14*(12), 2985-2994. <https://doi.org/10.1105/tpc.004630>
