# Supplementary material for: Guanidine production by plant homoarginine-6-hydroxylases
Source: eLife. 2024 Apr 15;12:RP91458. doi: 10.7554/eLife.91458 (PMC11018352; doi:10.7554/eLife.91458)
Supplement: Supplementary file 3. — Supplementary file 3 references: Alonso et al., 2003Kleinboelting et al., 2012Sessions et al., 2002 [file elife-91458-supp3.docx]

**Supplementary file 3: Sequences of primers used in this study**

| For cDNA insertion into pET24 (pET) or pENTR (pE) | |
| --- | --- |
| DIN11L-pET-f | gtactttcaaggtgctATGGTAATATATCATCGCAAAG |
| Din11s-pET-f | tgtactttcaaggtgctATGGTGACAGACTTCAAATCC |
| DIN11-pET-r | gtggtgctcgagtgcccataTTAACTGTTTTCCAC |
| DIN11L-pE-f | caggctttaaaggaacctATGGTAATATATCATCGCAAAG |
| DIN11s-pE-f | caggctttaaaggaacctATGGTGACAGACTTCAAATCC |
| DIN11-pE-r | gaaagctgggtctagataACTGTTTTCCACTAAATTTGC |
| 49630-pET-f | tgtactttcaaggtgctATGGCGACAAACTTCAAATCC |
| 49630-pET-r | ggtggtgctcgagtgctaacaTTAACTGTGTTCCACT |
| 49630-pE-f | caggctttaaaggaaccATGGCGACAAACTTCAAATCC |
| 49630-pE-NS-r | gaaagctgggtctagataACTGTGTTCCACTAAATTTTG |
| 50210-pET-f | tgtactttcaaggtgctATGGCGACGGACTTCAAGTCT |
| 50210-pET-r | ggtggtgctcgagtgccaTTACATAGCAAAGTTGGTC |
| 50210-pE-f | caggctttaaaggaaccatggcgacggacttcaag |
| 50210-pE-NS-r | gaaagctgggtctagatgCATAGCAAAGTTGGTCTGGA |
| Os_ODD33-pET-f | tgtactttcaaggtgctATGGGTTCTGACTTCAAGGC |
| Os_ODD33-pET-r | ggtggtgctcgagtgccaTTACATGACGAAGTTTGTCAAG |
| Gm_2-ODD-C23-pET-f | tgtactttcaaggtgctATGGCAACGGACTTTAGTTC |
| Gm_2-ODD-C23-pET-r | ggtggtgctcgagtgccaTTACAAATCAACAAAATTTGTGAGGACC |
| Ava_5009-pET-f | tgtactttcaaggtgcATGACAGTCTTACAACTTCCT |
| Ava_5009-pET-r | ggtggtgctcgagtgcctCTAAAGCACTTTTTGACG |
| ALDH7B4-pET-f | tgtactttcaaggtgctgctATGGGTTCGGCGAACAACGAG |
| ALDH7B4-pET-r | ggtggtgctcgagtgcCTAACCGAAGTTAATTCCTTGC |
| For Genotyping of T-DNA insertion lines | |
| Din11-f | tcaagggatttggatcactcac |
| Din11-r | tcgatcgaatgcatgcatcac |
| At3g46930-f | CTGCTGGATACAGGTTTATTCCAC |
| At3g46930-r | cacgacaatacccattaggctc |
| At3g49630-f2 | gcttgagtgatgtttccaagtag |
| At3g49630-r2 | gagggatggcaaaatcgtag |
| At3g50210-f | CACCATGGCGACGGACTTCAAGTCT |
| At3g50210-r | AGGTTGGCCATTTGTGAACTCAG |
| SALK-LB | TTCGGAACCACCATCAAACAG |
| GABI-LB | AATAACGCTGCGGACATCTAC |
| SAIL-LB | TACCAATACATTACACTAGCATCTG |
| For generation and detection of genome editing sites | |
| gRNA-f | attgCAGATTGGTCATGGAATAT |
| gRNA-r | aaacATATTCCATGACCAATCTG |
| Din11-HRM-f | CTGATTATGGTTGGTGAGTGTG |
| Din11-HRM-r | CAACGATCGTTGATATAGCAGGT |
| At3g49630-HRM-f | TTGATTTTGGTCGGTGATTGTT |
| At3g49630-HRM-r | AGCCATCTAATCTAACTAATCGTGA |

Lower case letters indicate overlaps with vector sequences for cloning
